# The Impact of Research Pressure on Depression Tendency among Chinese Doctoral Students: The Mediating Effect of Familial Financial Support

**DOI:** 10.3390/bs14080662

**Published:** 2024-08-01

**Authors:** Xiaoqing Xu, Guandong Song, Bin Xiao, Shuangjia Lin

**Affiliations:** 1 School of Humanities and Law, Northeastern University, Shenyang 110169, China; xxq@sjzu.edu.cn; 2 School of Management, Shenyang Jianzhu University, Shenyang 110168, China; 3 School of Educational Sciences, Bohai University, Jinzhou 121013, China; shuangjia_linbhu@163.com

**Keywords:** research pressure, depression tendency, familial financial support, mediating effect, doctoral students

## Abstract

Objective: This study aims to explore the impact of research pressure on depression tendency among Chinese doctoral students and analyze the mediating effect of familial financial support in this relationship. Methods: Based on the ecological systems theory, this study employs a mediating effect model and OLS regression model for empirical analysis. Through an online questionnaire, 2815 valid data from Chinese doctoral students were successfully collected. Conclusion: The study finds that research pressure has a significant positive impact on depression tendency among doctoral students (t = 18.347, *p* < 0.01). Married doctoral students show relatively lower depression tendency, indicating a negative impact of marital status on depression tendency (t = 12.579, *p* < 0.01). In terms of gender, female doctoral students are more prone to depression compared to their male counterparts (t = −2.921, *p* < 0.01). Additionally, as the doctoral year progresses, depression tendency also tends to increase (t = 3.690, *p* < 0.01). Importantly, familial financial support is proven to be a significant mediator between research pressure and depression tendency, explaining 32.116% of the relationship. Suggestion: This study not only provides a multi-dimensional perspective for understanding the mental health issues of doctoral students but also offers a scientific basis for universities and related educational departments to formulate more precise mental health intervention strategies.

## 1. Introduction

With the intensifying competition in scientific research and rising academic demands, doctoral students are increasingly experiencing mounting research pressure, which has been identified as a significant contributor to mental health issues, particularly depression. Meanwhile, mental health issues among doctoral students, particularly depression, have garnered significant attention due to their rising prevalence. Among the myriad of influencing factors, research pressure stands out as a key contributor to doctoral students’ mental health issues, as supported by numerous studies [1]. However, research pressure does not operate in isolation but rather interacts complexly with other factors, including familial financial support, which may serve as a buffer against its negative effects [2].

A plethora of international studies have consistently documented the high prevalence and severity of mental health issues, particularly depression, among doctoral students. For instance, a survey conducted by sociologist Katia Levecque and her colleagues among 3659 doctoral students in the Flemish Region of Belgium in 2017 showed that up to 32% of doctoral students are at risk of mental illness, with depression being the most prominent [3]. More shockingly, the results of a global survey of doctoral students conducted by Nature magazine in 2019 revealed that 36% of doctoral students had sought help for depression or anxiety issues, a significant increase from 12% in 2017 [4]. These statistics not only highlight the severity of mental health issues among doctoral students but also reflect the universality of this problem globally.

In China, the mental health challenges faced by doctoral students are no exception and warrant urgent attention. According to a special investigation on the mental health of graduate students in the “Report on the Development of Mental Health in China (2019–2020)”, recently released by the Institute of Psychology of the Chinese Academy of Sciences, 35.5% of graduate students may show some degree of depression [5]. Since doctoral students are a subset of the graduate student population, these data pertain to the depression problems of Chinese doctoral students, further highlighting the urgency of this issue [6]. It is worth noting that the report also points out that the average level of depression and anxiety among female students is higher than that of male students, increasing the attention paid to the mental health of this particular group.

Research pressure, an inevitable aspect of doctoral studies, has been closely linked to mental health issues, particularly depression, as evidenced by numerous studies [7]. This pressure stems from the pursuit of academic achievements, rigid requirements such as graduation and paper publication, and is also related to doctoral students’ identification with their self-worth, uncertainty about future career development, and expectations from family and society [8]. When these pressures accumulate and exceed the coping abilities of doctoral students, it may trigger mental health issues such as depression.

In this context, the significance of familial financial support emerges as a potential mitigating factor in the relationship between research pressure and mental health issues. For doctoral students, familial financial support is not only the economic foundation of their academic pursuits but also their psychological reliance when facing pressure [9,10]. When doctoral students are under research pressure, economic and emotional support from their families can help them better cope with challenges and alleviate negative emotions, thereby reducing the risk of depression [11]. However, we should also recognize that familial financial support is not a panacea for solving all problems. Its mechanism of action and actual effects may vary due to individual differences, family environments, social backgrounds, and other factors.

In this context, research pressure can potentially impact family financial support in multifaceted ways. Firstly, research pressure can lead to increased financial stress within doctoral students’ households [1]. As doctoral students dedicate more time and energy to their studies, they may have limited opportunities for part-time work or other income-generating activities [2]. This reduction in income, coupled with the rising costs associated with pursuing higher education, can put strain on family finances. Families who perceive their doctoral student as under significant research pressure may, therefore, adjust their financial support accordingly, either out of necessity or a belief that additional financial resources could alleviate some of the stress. Secondly, research pressure can influence the perception and expectations of families towards their doctoral students [12]. High expectations from academic institutions, advisors, and society at large can translate into additional pressure on doctoral students to perform well and achieve their goals. When these expectations are not met, or progress seems slow, families may feel discouraged or uncertain about the future prospects of their doctoral student [13]. Such sentiments could, in turn, affect the level of financial support they are willing or able to provide [14].

Drawing upon the ecosystem theory, we adopt a holistic approach to understand the intricate interplay between individual, family, and external factors that contribute to doctoral student depression [15]. The ecological circle, originally proposed by ecologist and botanist Arthur Tansley in 1935, refers to an integrated system that includes organisms and their living environment [16]. In this system, different elements (including biological and non-biological components) interact through material cycling, energy flow, and information exchange, forming an indivisible natural community. This theory has applications in economics, psychology, and information theory [17]. Under this theoretical framework, doctoral student depression is not just an individual psychological issue but rather the result of interactions between multiple levels, such as individual, family, and external pressure. This theoretical framework places the individual (doctoral student) within a multifaceted ecosystem for examination [18]. This ecosystem encompasses multiple interconnected and influencing levels, including the micro (individual level), meso (family level), and macro (external pressure level) [19,20,21].

Individual Level: This focuses on the psychological state of the individual, such as depressive tendencies among doctoral students, which is a direct reflection of their mental health. An individual’s psychological state is influenced by various factors, including personal traits, cognition, and emotions.

Family Level: Familial financial support serves as a crucial meso-level factor that significantly impacts an individual’s psychological state. The quality of a family’s economic conditions directly relates to an individual’s quality of life and psychological stress, thereby affecting their mental health.

**Figure 1:**
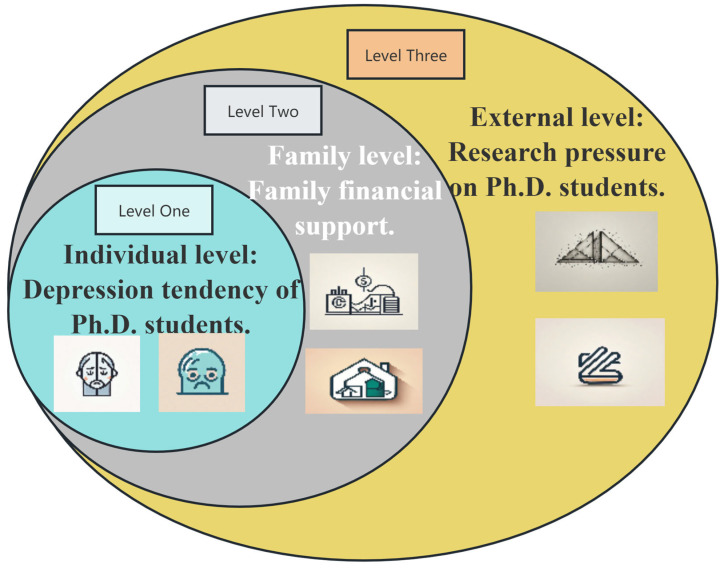
Research construct based on ecosystem theory.

External Pressure Level: Academic research pressure is one of the primary macro-level pressures faced by doctoral students. This pressure not only stems from academic requirements but is also influenced by factors such as career development and societal expectations. The magnitude of academic research pressure directly impacts the psychological state of doctoral students, and excessive pressure may lead to mental health issues like depression (Figure 1). Moreover, high research pressure can indirectly affect the use of family financial support as students, under stress, may seek additional financial resources to alleviate the burden of their studies or to cope with the demands of their research, thereby increasing reliance on family support.

Within this theoretical framework, the individual, family, and external pressure levels are interconnected and mutually influential, collectively forming a dynamic ecosystem. When doctoral students face academic research pressure, their psychological state is affected, and this impact can potentially ripple into the family level, altering the perception and utilization of familial financial support. Simultaneously, the status of familial financial support, in turn, influences the doctoral students’ ability to cope with academic research pressure and their psychological state.

In view of this, this article will comprehensively use quantitative and qualitative research methods to deeply analyze the complex relationship between research pressure, familial financial support, and depressive tendencies among doctoral students. Through means such as questionnaire surveys, in-depth interviews, and data analysis, we hope to comprehensively and objectively reveal the intrinsic connection between these three aspects.

## 2. Research Review and Theoretical Basis

### 2.1. Scientific Research Pressure and Depression Tendency

There is a close connection between scientific research pressure and depression tendency among doctoral students, which is supported by the transactional theory of stress and coping [22]. According to this theory, psychological stress arises when the environmental demands faced by an individual exceed their coping abilities [15]. If such stress persists and cannot be effectively relieved, it may lead to mental health issues, including depression [23]. For doctoral students, as scientific research tasks intensify and academic expectations increase, the pressure they face also grows. The significant correlation between this pressure and the emergence of depressive symptoms has been confirmed by multiple studies [1,24].

The negative impact of scientific research pressure on the mental health of doctoral students can be explained from multiple dimensions. Firstly, according to the Conservation of Resources Theory (COR), people always strive to acquire, protect, and retain resources, and psychological stress arises when these resources are threatened or lost [25]. The scientific research pressure faced by doctoral students may make them feel a lack of resources such as time and energy, leading to psychological stress and depressive emotions. Secondly, long-term exposure to high-intensity scientific research tasks may cause doctoral students to fall into a vortex of “over-effort”, resulting in physical and mental exhaustion and increasing the risk of depression [26]. Finally, scientific research pressure may also exacerbate a depression tendency by affecting doctoral students’ self-efficacy. Self-efficacy refers to an individual’s belief in their ability to complete a certain task [27]. When doctoral students face tremendous scientific research pressure, they may doubt their abilities, thereby reducing self-efficacy and increasing the risk of depression. Multiple studies have shown that as scientific research tasks intensify and academic expectations increase, the pressure faced by doctoral students also grows. This pressure has a significant correlation with the emergence of depressive symptoms [1,24]. Scientific research pressure not only stems from the pursuit of academic achievements but also includes multiple factors such as competition with peers, mentors’ expectations, and experimental failures. Long-term scientific research pressure may lead to depressive symptoms such as low mood, reduced self-worth, loss of interest and pleasure among doctoral students, thereby affecting their academic and life quality [2].

**Hypothesis** **1.** *There is a positive correlation between doctoral students* *’ scientific research pressure and their depression tendency; that is, the greater the scientific research pressure, the higher the depression tendency of doctoral students.*

### 2.2. The Importance of Familial Financial Support

Familial financial support for PhD students is not only material assistance but also emotional comfort. The significance of familial financial support for PhD students can be explained from the perspective of social support theory, as Cohen and Wills suggested that an individual’s social environment can provide emotional, informational, and material support, helping them better cope with stress and challenges [28]. For PhD students, familial financial support, as Rospenda et al. pointed out, is not just material assistance; it also represents emotional support and recognition, creating a relatively stable and carefree learning environment and enabling students to focus more on their research [29].

From an empirical perspective, the positive impact of familial financial support on the mental health of PhD students has been widely documented. Eisenberg et al. found that familial financial support can alleviate PhD students’ financial pressure, reducing negative emotions such as anxiety and depression caused by economic difficulties [30]. More importantly, as Becker et al. emphasized, familial financial support signifies familial recognition and encouragement for PhD students’ academic pursuits, and this psychological support plays a crucial role in enhancing PhD students’ self-confidence and resilience [31]. In other words, as Rospenda et al. reiterated, familial financial support not only provides material security for PhD students but also offers them emotional comfort and motivation, creating a relatively stable and carefree learning environment and allowing them to focus on their research [32]. Again, Eisenberg et al. demonstrated that familial financial support reduces PhD students’ financial pressure and negative emotions like anxiety and depression due to financial difficulties [33]. Additionally, Becker et al. showed that familial financial support represents the family’s acknowledgment and encouragement of the student’s academic pursuits, bolstering their self-confidence and resilience [34].

**Hypothesis** **2.** *There is a negative correlation between familial financial support and depression tendency among PhD students, meaning that higher familial financial support leads to a lower depression tendency among PhD students.*

### 2.3. The Mediating Effect of Familial Financial Support

Familial financial support may play a crucial mediating role between research pressure and depression tendency. This viewpoint is supported by the Stress-Buffering Hypothesis, which suggests that social support can buffer the negative impact of stress on mental health [23]. For PhD students, familial financial support, as an important form of social support, may exert a mediating effect by mitigating the negative impact of research pressure on mental health.

Specifically, research pressure increases the risk of depression by affecting the psychological state of PhD students, while familial financial support can serve as a buffering mechanism to alleviate this psychological pressure [35]. Adequate familial financial support allows PhD students to face difficulties and challenges in research more calmly, reducing additional pressure arising from economic concerns. Simultaneously, it implies that PhD students have more resources and choices, enabling them to better cope with and adapt to various changes and challenges in academic life [36]. Research pressure increases depression risk by affecting PhD students’ psychological states, while familial financial support acts as a buffering mechanism to ease this psychological burden [35]. With sufficient familial financial support, PhD students can face the difficulties and challenges of research more calmly, reducing the extra pressure caused by financial worries [37]. Additionally, it signifies that PhD students possess more resources and options, enhancing their ability to handle and adjust to diverse changes and challenges in academic life [36]. Thus, we can reasonably speculate that familial financial support plays a mediating role between research pressure and depression tendency.

**Hypothesis** **3.** *Familial financial support mediates the relationship between research pressure and depression tendency, meaning that increased familial financial support decreases PhD students* *’ depression tendency created by research pressure.*

In summary, there are complex relationships among research pressure, familial financial support, and depression tendency in PhD students. By delving deeper into these relationships, we can provide more targeted support and assistance to PhD students, thereby reducing their depression risk and enhancing the quality of their academic and personal lives.

## 3. Research Methods and Survey Design

This study aims to delve into the relationship between research pressure, familial financial support, and depression tendency among doctoral students. To achieve this research objective, we adopted a combination of quantitative and qualitative research methods, specifically including the following steps:

### 3.1. Scale Selection

**(1) Research Pressure Scale**: To accurately assess the research pressure of doctoral students, we utilized a self-designed Research Pressure Scale [28]. This scale consists of 10 items, with each item rated on a 5-point scale (1 = no pressure at all, 5 = extreme pressure). It aims to measure doctoral students’ stress levels in areas such as research tasks, academic expectations, and time management.

Reliability Test Results: The reliability of the scale was assessed through internal consistency, with Cronbach’s Alpha being the evaluation metric. In our pretest, the Cronbach’s Alpha value of the scale was 0.85, indicating good internal consistency reliability. This suggests that the items on the scale measure the same underlying concept consistently.

Validity Test Results: Content Validity: To ensure the scale’s content validity, we invited several experts in related fields to review the items. Their feedback helped ensure that the scale comprehensively covers various aspects of research pressure experienced by doctoral students.

Construct Validity: Construct validity was evaluated using factor analysis. The results supported the dimensional structure of the scale, indicating that it effectively captures the construct of research pressure among doctoral students.

**(2) Family Economic Support Scale**: To measure the impact of family economic support on doctoral students, we used an 8-item Family Economic Support Scale [29]. This scale employs a 5-point scoring system (1 = no support at all, 5 = full support) to assess the level of support doctoral students receive from their families in terms of finance, emotion, and academic resources.

Reliability Test Results: The internal consistency reliability of this scale was evaluated using Cronbach’s Alpha. In our study, the Cronbach’s Alpha value exceeded 0.80, demonstrating good reliability. This indicates that the scale items consistently measure the construct of family economic support, suggesting high stability and dependability in the measurements.

Validity Test Results: Content Validity: To ensure content validity, we had several experts in related fields review the scale items. Their feedback helped confirm that the scale items comprehensively reflect different aspects of family economic support, including financial assistance, emotional support, and academic resources.

Construct Validity: Construct validity was evaluated using factor analysis. The results supported the dimensional structure of the scale, confirming its effectiveness in measuring family economic support among doctoral students.

**(3) Depression Tendency Scale**: To assess depression tendency among doctoral students, we selected the widely used Self-Rating Depression Scale (SDS) [30]. This scale consists of 20 items, rated on a 4-point scale (1 = occasionally, 4 = always), effectively measuring individual depressive symptoms and their severity.

Reliability Test Results: The reliability of the SDS has been validated in multiple studies. In our sample, the Cronbach’s Alpha value exceeded 0.90, indicating excellent internal consistency reliability. This demonstrates that the items on the scale consistently measure depressive symptoms, ensuring stable and reliable results.

Validity Test Results: Content Validity: The SDS has undergone extensive clinical validation, demonstrating good content validity. The scale items cover a broad range of depressive symptoms, ensuring comprehensive assessment.

Construct Validity: Construct validity has also been well established through numerous studies. In our study, we further confirmed the effectiveness of the SDS through correlation analysis with other mental health scales, indicating its ability to accurately reflect depression tendency among doctoral students.

### 3.2. Research Design

**(1) Geographic and University Selection**: To ensure the breadth and representativeness of the data, we selected seven provinces in South China, North China, and Central China, covering different geographical, economic, and cultural backgrounds. Additionally, we chose 20 universities with doctoral programs as research subjects, which are representative in terms of academic fields, faculty strength, and scientific research level.

**(2) Questionnaire Design and Production**: We integrated the three scales of research pressure, family economic support, and depression tendency to form a complete survey questionnaire. The questionnaire is designed to be concise and clear, with clear problem statements and reasonable scoring methods to ensure the objectivity and accuracy of the data.

**(3) Questionnaire Distribution and Collection**: From January 2023 to January 2024, we distributed a total of 2896 questionnaires to doctoral students in target universities. The questionnaires were distributed through various channels such as email, social media, and the university’s internal online survey system to ensure coverage and response rates. Ultimately, we successfully collected 2815 valid data points, achieving a high response rate of 97.2%, providing a solid foundation for subsequent data analysis.

**(4) Data Cleaning and Organization**: During the data cleaning phase, we strictly eliminated invalid questionnaires and outliers to ensure data authenticity and validity. The criteria for deeming questionnaires invalid included:

Incomplete responses.

Identical responses for all items, indicating lack of engagement.

Extremely short response times that suggest the questionnaire was not filled out thoughtfully.

The data cleaning steps were as follows:

Step 1: Initial screening to remove incomplete questionnaires.

Step 2: Examination of response patterns to identify and exclude questionnaires with identical responses for all items.

Step 3: Analysis of response times to exclude questionnaires completed in an unrealistically short amount of time.

Step 4: Identification and removal of statistical outliers using standard deviation and interquartile range (IQR) methods to ensure that extreme values do not skew the results.

### 3.3. Data Analysis Methods

We first conducted descriptive statistical analysis to understand the overall situation of doctoral students in terms of research pressure, family economic support, and depression tendency. Subsequently, correlation analysis was used to explore the relationship between various variables. Finally, a structural equation model was employed to verify the mediating effect of family economic support between research pressure and depression tendency.

**(1) Descriptive Statistics**: Firstly, the latest version of SPSS (Statistical Package for the Social Sciences), SPSS 28.0, was used to perform descriptive statistical analysis on the collected data, including mean values, standard deviations, etc., to gain insights into the overall situation of doctoral students regarding research pressure, family economic support, and depression tendency.

**(2) Correlation Analysis**: Next, we explored the relationships among research pressure, family economic support, and depression tendency through correlation analysis, verifying the positive and negative correlations postulated in our research hypotheses.

**(3) Mediation Effect Analysis**: To examine the mediating effect of family economic support between research pressure and depression tendency, we utilized Structural Equation Modeling (SEM) for analysis, specifically using Amos 26.0. By constructing and testing the model’s path coefficients, we could clarify the mechanism of family economic support within this relationship.

## 4. Research Results

### 4.1. Descriptive Statistical Analysis

To better present the demographic and basic characteristics of doctoral students in this study, the following table shows the results of descriptive statistical analysis. The data are categorized in detail, and the number and percentage of each category are calculated to provide a more comprehensive portrait of the doctoral student population (Table 1).

Firstly, in terms of the type of doctoral degree being pursued, the number of Engineering Doctorate (EngD) and Doctor of Science (DSc) students is relatively high, accounting for 19.54% and 15.27%, respectively. Although there are also a certain number of doctorates in other fields, such as law, economics, education, and medicine, their proportions are relatively small in comparison. This indicates that research in engineering and science fields, specifically among doctoral students, appears to be relatively more concentrated. However, whether this observation holds true for China as a whole or specifically within my sample requires clarification. If referring to China, it is noteworthy that the majority of doctoral students in these fields are actively engaged in research, with a strong emphasis on STEM disciplines, including engineering and science. Alternatively, if discussing my sample, it would depend on the specific demographics and research interests of the doctoral students included therein. In terms of gender distribution, the proportion of males and females is roughly equal, with males slightly predominating, accounting for 53.29% of the total and females accounting for 46.71%. These data reflect that gender balance is gradually improving in high-level academic pursuits. Regarding the doctoral students’ backgrounds, there are slightly more students from urban areas (56.85%) than those from rural areas (43.15%). This may suggest the influence of urban and rural education resource allocation or family background on students’ pursuit of doctoral degrees. In terms of grade distribution, doctoral students from the first year to the sixth year and beyond account for a certain proportion, with a higher proportion of students in the first and sixth years and beyond, accounting for 19.54% and 23.27%, respectively. This may indicate that the early and late stages of doctoral education are critical phases of research. Regarding the annual household income of doctoral students, the data reveal a distribution across various income ranges. Notably, the highest proportion (24.87%) of families earn between CNY 100,000 and 300,000 annually. In the context of China, this income range is typically considered to represent high-income households. These data provide a certain reference for understanding the economic status and family background of doctoral students. Finally, in terms of marital status, there are slightly more unmarried doctoral students (53.81%) than married ones (46.19%). These data suggest that, while many students may choose to prioritize their studies during their doctoral program, a significant portion of the population, almost half, is married, indicating that marital status is not necessarily a secondary consideration for all students.

In summary, this table depicts a comprehensive and three-dimensional portrait of the doctoral student population through multi-dimensional demographic and academic characteristic data.

### 4.2. Direct Effect Test

After conducting descriptive statistical analysis, this study performed a direct effect test on relevant variables. Table 2 details multiple variables of doctoral students, their types, statistical indicators, and correlations between three key indicators: research pressure, family financial support, and depression tendency. The following is an in-depth description and analysis of the table content:

Firstly, we notice that the table covers various types of variables, including categorical variables (such as doctoral degree type, gender, and marital status) and continuous variables (such as grade, annual household income).

Secondly, from the perspective of correlation analysis, the correlation coefficients between various variables and research pressure, family financial support, and depression tendency are presented. These coefficients reveal potential relationships between these variables. The correlation between the type of doctoral degree and research pressure, family financial support, and depression tendency is not significant. This may be because the doctoral degree itself does not directly determine these psychological and social factors. In terms of gender, we can see that female doctoral students have significantly higher research pressure and depression tendency than male doctoral students. As the duration of doctoral studies increases, students’ research pressure increases, family financial support weakens, and depression tendency is higher. In addition, doctoral students from families with higher annual incomes have lower research pressure and depression tendency and higher family financial support.

Finally, the data reveal a slight negative correlation between marital status and both research pressure and depression tendency among doctoral students. This observation suggests that married doctoral students might experience lower levels of research pressure and depression. One possible explanation for this correlation could be that married students may receive additional financial and psychological support from their spouses. This support system could potentially alleviate some of the stress and pressure associated with their research, ultimately contributing to a reduced depression tendency.

In summary, the following table reveals the characteristics of doctoral students in different aspects and their relationships with key indicators through detailed data and correlation analysis. These findings have important guiding significance for deeply understanding the academic and living environments of doctoral students and providing them with better support and services.

To further investigate the direct effect relationship between research pressure, depression tendency, and family financial support, the author conducted an additional analysis. Table 3 provides the mean and standard deviation of research pressure, family financial support, and depression tendency and displays the correlations between them. The following is an academic interpretation of the table:

Firstly, research pressure. The mean value is 3.45, and the standard deviation is 0.84, indicating that doctoral students in the sample generally face a certain level of research pressure, and there is some variability. The correlation between research pressure and depression tendency is 0.015, with a *p*-value less than 0.05, indicating a significant positive correlation between research pressure and depression tendency. The correlation between research pressure and family financial support is −0.020, with a *p*-value less than 0.05, indicating a significant negative correlation between research pressure and family financial support.

Secondly, family financial support. The mean value is 2.86, and the standard deviation is 0.74, indicating that the level of family financial support for doctoral students in the sample is generally low, and there is some variability. The correlation between family financial support and depression tendency is −0.025, with a *p*-value less than 0.01, indicating a significant negative correlation between family financial support and depression tendency.

Thirdly, depression tendency. The mean value is 3.65, and the standard deviation is 0.71, indicating that doctoral students in the sample generally have a certain degree of depression tendency, and there is some variability.

Taken together, these results demonstrate the correlations between research pressure, family financial support, and depression tendency. The direct effect results pave the way for mediation effect testing. In particular, the significant correlation between research pressure and depression tendency, as well as family financial support, suggests the necessity of measures to reduce research pressure and increase family financial support in improving the mental health and academic support of doctoral students.

### 4.3. Mediation Effect Test

#### 4.3.1. Linear Regression Model

Multiple regression analysis was used as the research method, employing SPSS 28.0 software. The dependent variable was depression tendency, while the independent variable was research pressure. Control variables included place of origin, total household income, gender, and others. Statistical significance was determined by *p* < 0.05. Using OLS regression allowed for a clear representation of the relationship between the dependent and independent variables, determining significance (Table 4).

In this study, the research pressure indicator was first used as an independent variable for OLS regression analysis, and the robust standard error regression method was used for this study. As can be seen from the table below, the R-squared value of the model is 0.642, which means that this indicator can explain 64.24% of the variation in depression tendency. It was found that the model passed the F-test (F = 165.010, *p* = 0.000 < 0.05). Furthermore, research pressure had a significant positive association with depression tendency (t = 18.347, *p* = 0.000 < 0.01). Marital status had a significant positive association with depression tendency (t = 12.579, *p* = 0.000 < 0.01). Annual household income had a significant negative association with depression tendency (t = −2.119, *p* = −0.034 < 0.05). Gender had a significant negative impact on depression tendency (t = −2.921, *p* = −0.003 < 0.01). PhD grade had a significant positive association with depression tendency (t = 3.690, *p* = 0.000 < 0.01).

#### 4.3.2. Mediation Effect Test

##### Path Analysis

**Figure 2:**
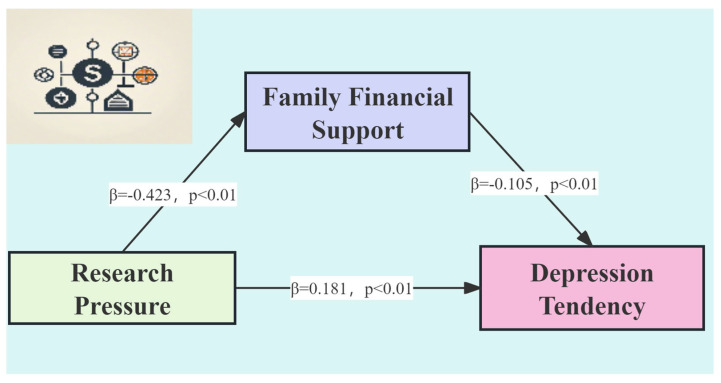
Simplified diagram of path analysis.

This study analyzed the influence paths of the structural equation model and found that the model’s goodness-of-fit indicators were excellent, with all influence paths in the model meeting statistically significant criteria (Figure 2). Research pressure had a significantly negative association with household economic support (β = −0.423, *p* < 0.01), and household economic support had a significantly negative relationship with depression tendency (β = −0.105, *p* < 0.01). Additionally, research pressure had a significantly positive association with depression tendency (β = 0.181, *p* < 0.01).

##### Mediation Effect Test

Researchers conducted an in-depth analysis of the mediation effect variables tested in this study using SPSS software and employed the Bootstrap sampling method to test the mediation effect. According to the mediation model, the F-statistic is used to evaluate the significance of the regression model to determine whether the entire model has statistical significance. Regarding the relationship between research pressure and depression tendency (F(1,2498) = 17.643, *p* = 0.000), the results are significant, indicating a significant linear relationship between research pressure and depression tendency. Similarly, the relationship between household economic support and depression tendency (F(2,2497) = 17.839, *p* = 0.000) is also highly significant. These results suggest that both research pressure and household economic support have a significant association with depression tendency.

Furthermore, the mediation effect analysis involves three models, as follows:

Depression Tendency = 7.122 + 0.181 Research Pressure

Household Economic Support = 1.283 − 0.423 Research Pressure

Depression Tendency = 7.257 + 0.137 Research Pressure − 0.105 Household Economic Support

Based on the above models and combined with the results in Table 5, the researchers draw the following conclusions: Research pressure has a positive predictive effect on depression tendency (f1 = 0.181, *p* < 0.01). After controlling for the influence of depression tendency, research pressure has a negative predictive effect on household economic support (f2 = −0.423, *p* < 0.01); furthermore, after controlling for the influence of research pressure, household economic support has a negative predictive effect on depression tendency (f3 = −0.105, *p* < 0.01).

To determine whether the mediation effect of household economic support is significant, this study employed a bias-corrected nonparametric percentile Bootstrap sampling method in SPSS software. By repeatedly and randomly sampling 1000 samples from the population, researchers obtained 95% confidence intervals (CI) for direct, indirect, and total effects to assess the significance of the effect. The test results showed that this mediation effect is a partial mediation effect (Table 6). This means that research pressure exerts a mediation effect on depression tendency through household economic support, and this effect is significant, accounting for 32.116% of the total effect.

## 5. Research Conclusions

### 5.1. Significant Association between Research Pressure and Depression Tendency

Through empirical analysis, this study found a significant and positively correlated impact of research pressure on the depression tendency of PhD students (t = 18.347, *p* = 0.000 < 0.01), which cannot be ignored. In a high-intensity academic research environment, PhD students face particularly heavy psychological loads, mainly stemming from the arduous nature of research tasks, the excessive pursuit of high-level academic achievements, and the complexity of time allocation and management [31]. These factors intertwine, forming a potential risk network that induces depressive emotions.

Compared to previous studies, our results further validate the close connection between research pressure and mental health and reveal unique stressors in the university environment. It is worth noting that although academic research inherently requires a certain amount of pressure and challenges to drive knowledge innovation and progress, excessive pressure can negatively impact researchers’ mental health [32].

From a theoretical perspective, this finding aligns with stress coping theory, which suggests that long-term, high-intensity stress, if not effectively coped with, can lead to mental health issues. Therefore, while emphasizing academic achievements, universities and educational institutions should also focus on the mental health of PhD students [33]. By providing supportive resources such as psychological counseling and stress management training, a more balanced and healthy academic ecological environment can be built. This not only contributes to the comprehensive development of individual PhD students but also holds significant importance for enhancing the overall quality of academic research [35].

### 5.2. The Relationship between Marriage, Gender, Grade, and Depression Tendency

Interestingly, married PhD students tend to have a relatively lower depression tendency [36]. This may be related to the emotional support and stable social relationships that married PhD students can obtain from their spouses. This kind of support may help alleviate academic and life pressures, thereby reducing the risk of depression. This finding underscores the importance of social support, especially support from spouses, in maintaining mental health [34].

Meanwhile, gender has a significant negative association with depression tendency (t = −2.921, *p* = 0.003 < 0.01), indicating that female PhD students may be more susceptible to depression. In addition, PhD grade also has a significant positive impact on depression tendency (t = 3.690, *p* = 0.000 < 0.01). As academic studies progress and graduation pressure increases, the risk of depression among senior PhD students may rise accordingly.

These findings suggest that when focusing on the mental health of PhD students, their personal characteristics and living environments should be fully considered.

### 5.3. Negative Correlation between Family Economic Status and Depression Tendency

It is worth noting that annual household income has a significant negative association with depression tendency (t = −2.119, *p* = 0.034 < 0.05). This suggests that PhD students from lower-income families may face a higher risk of depression. This could be related to economic pressure, inadequate social support, and the resulting feelings of inferiority and anxiety. Therefore, when providing economic assistance, universities should pay special attention to PhD students from low-income families, ensuring that they receive sufficient support to complete their studies and maintain good mental health. At the same time, strengthening home-school cooperation and providing services such as career planning and employment guidance can also help them better cope with future challenges [37].

In summary, this study reveals the influence of various factors on the depression tendency of PhD students. To reduce the risk of depression among PhD students, universities and society should provide comprehensive support and resources, with special attention to female PhD students, senior PhD students, and those from economically disadvantaged backgrounds [38,39]. While it is beneficial for PhD students to establish stable social relationships, including marital relationships, for emotional support, it is acknowledged that this may not always be feasible, especially for students living in new cities or facing other challenges.

### 5.4. Results of the Mediating Effect of Familial Financial Support

This study delved into the impact of research pressure on the depression tendency of Chinese PhD students, with a particular focus on the mediating role of familial financial support. Through a carefully constructed model and rigorous empirical analysis, we draw the following academically valuable conclusions:

Firstly, research pressure significantly positively predicts the depression tendency of PhD students. This result echoes previous studies, further reinforcing the theoretical foundation that research pressure has a negative association with mental health. This highlights the importance of mental health issues among PhD students in a high-pressure academic environment, which cannot be ignored [40,41].

Secondly, after controlling for variables related to depression tendency, this study observed a significant negative predictive effect of research pressure on familial financial support. This finding suggests another factor that may be associated with changes in financial support over time [42]: as research tasks increase, PhD students may face a reduction in familial financial support. This reflects the potential conflict between research and family life, especially in terms of time allocation and resource management.

More importantly, after stripping away the influence of research pressure, we found that familial financial support has a significant negative predictive effect on depression tendency [43]. This indicates that stable familial financial support not only provides material security for PhD students but also gives them a sense of safety and stability at the psychological level, which is crucial for reducing the risk of depression.

To verify the robustness of these findings, we adopted rigorous statistical methods and randomly sampled 1000 individuals from the population for repeated testing. By calculating the 95% confidence interval (CI) for the direct, indirect, and total effects, we confirmed the partial mediating effect of familial financial support between research pressure and depression tendency. This effect is significant, accounting for a high proportion of 32.116%. This result confirms our hypothesis and provides a solid foundation for further theoretical construction and empirical research to explore this relationship more deeply.

In summary, this study not only reveals the close connection between research pressure and depression tendency but also deeply analyzes the important role of familial financial support in this relationship. These findings have important theoretical and practical implications for understanding the influencing factors of PhD students’ mental health and formulating effective intervention strategies.

## 6. Research Limitations

Sample Representativeness: The sample of this study may not be comprehensive enough, mainly focusing on PhD students from a specific region or university. Therefore, it may not fully reflect the real situation of PhD students nationwide. Future research needs to expand the sample scope to improve the external validity of the study.

Cross-sectional Study Design: This study employs a cross-sectional research design, which inherently limits its ability to establish temporal precedence or directionality between variables. Consequently, the associations observed between factors such as research pressure, familial financial support, and depression tendency should be interpreted cautiously, as they do not necessarily indicate cause-and-effect relationships.

Limitations of Measurement Tools: The scales used in this study may have a certain degree of subjectivity and may not cover all relevant psychological and social factors. Future research can consider developing or introducing more objective and comprehensive measurement tools to improve the accuracy and reliability of the study.

## 7. Research Prospects

Future research can delve deeper into the multifaceted formation mechanism of depression tendency among PhD students by investigating additional potential influencing factors, including academic environment, mentorship style, and personal personality traits. This comprehensive approach will contribute to a more nuanced understanding of the intricate interplay among these factors and their roles in shaping PhD students’ mental health outcomes.

Research on Intervention Strategies: Based on the results of this study, targeted intervention strategies can be designed and implemented in the future, such as providing psychological counseling, financial assistance, mentor training, etc., to reduce the risk of depression among PhD students, and to evaluate the effectiveness of these interventions.

Interdisciplinary Cooperation: We encourage interdisciplinary cooperation among psychology, education, sociology, and other disciplines to deeply study the mental health issues of PhD students. By integrating theories and methods from different disciplines, the complexity and diversity of depression tendency among PhD students can be more fully revealed.

## Figures and Tables

**Table 1:** Demographic and academic characteristics of doctoral students.

Category	Subcategory	Quantity	Percentage	Description
**PhD Degrees**	Law PhD	300	10.66%	Type of degree pursued by doctoral students
	Economics PhD	450	15.99%	
	Education PhD	280	9.95%	
	Engineering PhD	550	19.54%	
	Science PhD	430	15.27%	
	Medical PhD	350	12.43%	
	Other Degrees	455	16.16%	
**Gender**	Male	1500	53.29%	Gender of doctoral students
	Female	1315	46.71%	
**Origin**	Urban	1600	56.85%	Place of origin of doctoral students
	Rural	1215	43.15%	
**Grade**	First Year PhD	550	19.54%	Current stage of study for doctoral students
	Second Year PhD	480	17.05%	
	Third Year PhD	420	14.92%	
	Fourth Year PhD	380	13.50%	
	Fifth Year PhD	330	11.72%	
	Sixth Year and Above PhD	655	23.27%	
**Annual Household Income**	10,000–30,000 RMB	450	15.99%	Annual income level of doctoral students’ families
	30,000–50,000 RMB	630	22.38%	
	50,000–100,000 RMB	580	20.59%	
	100,000–300,000 RMB	700	24.87%	
	300,000–1,000,000 RMB	455	16.16%	
**Marital Status**	Married	1300	46.19%	Marital status of doctoral students
	Unmarried	1515	53.81%	

**Table 2:** Correlation analysis between multi-dimensional characteristics of doctoral students and psychological and economic support.

Variable	Research Pressure	Family Financial Support	Depression Tendency
**PhD Degrees**	r = 0.12 (*p* = 0.279)	r = 0.09 (*p* = 0.375)	r = 0.13 (*p* = 0.268)
**Gender**	r = 0.68 (*p* = 0.075) *	r = 0.13 (*p* = 0.65)	r = 0.59 (*p* = 0.072) *
**Grade**	r = 0.63 (*p* = 0.057) *	r = 0.56 (*p* = 0.054) *	r = 0.64 (*p* = 0.065) *
**Annual Household Income**	r = −0.78 (*p* = 0.017) **	r = −0.70 (*p* = 0.013) **	r = −0.72 (*p* = 0.021) **
**Marital Status**	r = −0.57 (*p* = 0.051) *	r = 0.16 (*p* = 0.411)	r = −0.77 (*p* = 0.015) **

Note: *p* < 0.1, *; *p* < 0.05, **. PhD Degree: Represents the professional field of doctoral students, which is a categorical variable and has been coded for analysis. Gender: 0 represents female, 1 represents male, which is a categorical variable and has been coded for analysis. Grade: Is a continuous variable. Annual Household Income: Is a continuous variable. Marital Status: 0 represents unmarried, 1 represents married, which is a categorical variable and has been coded for analysis.

**Table 3:** Correlation analysis between research pressure, family financial support, and depression tendency.

Variable	Mean	Standard Deviation	Depression Tendency	Family Financial Support
**Research Pressure**	3.45	0.84	0.015 **	−0.020 **
**Family Financial Support**	2.86	0.74	−0.025 ***	-
**Depression Tendency**	3.65	0.71	-	-

Note: *p* < 0.05, **; *p* < 0.01, ***.

**Table 4:** OLS regression analysis results of the impact of scientific research pressure on depression tendency.

	Coef	Std.Err	t	*p*	95% CI	R^2^	F
**Constant**	17.443	1.311	13.308	0.000 **	14.874~20.012	0.642	F(7,331) = 165.010, *p* = 0.000
**Research Pressure**	−0.275	0.015	18.347	0.000 **	0.304~0.246		
**Marital Status**	−6.020	0.479	12.579	0.000 **	4.958~5.082		
**Grade**	−0.253	0.222	−1.142	0.254	−0.688~0.182		
**PhD Degree**	0.231	0.317	0.728	0.467	−0.391~0.853		
**Annual Household Income**	0.504	0.238	−2.119	−0.034 *	−0.188~−0.069		
**Gender**	0.885	0.303	−2.921	−0.003 **	−0.291~−0.178		
**PhD Year**	1.171	0.317	3.690	0.000 **	0.549~1.793		

Note: *p* < 0.1, *; *p* < 0.05, **. D-W: 0.569.

**Table 5:** Analysis results of the mediation model of household economic support (dependent variable: depression tendency).

	Depression Tendency	Family Financial Support	Depression Tendency
**Constant**	7.122 ** (38.730)	1.283 ** (16.721)	7.257 ** (25.612)
**Research Pressure**	0.181 ** (4.100)	−0.423 ** (3.067)	0.137 ** (3.155)
**Family Financial Support**			−0.105 ** (3.959)
**Sample size**	2518	2518	2518
***R* ^2^**	0.154	0.088	0.271
**Adjusted *R*^2^**	0.144	0.079	0.254
***F* Value**	*F*(1,2498) = 17.643, *p* = 0.000	*F*(1,2498) = 9.539, *p* = 0.003	*F*(2,2497) = 17.839, *p* = 0.000

Note: *p* < 0.05, **.

**Table 6:** Analysis of the results of the Bootstrap significance test for the mediated effect.

Item	c Total Effect	a	b	Mediation Effect a*b	(Boot SE) a*b	(z Value) a*b	(*p* Value) a*b	a*b (95% BootCI)	c’ Direct Effect	Conclusion	Proportion
**Research pressure => Family financial support => Depression tendency**	0.181 **	−0.423 **	−0.105 **	0.044	0.065	0.795	0.000	0.012~0.376	0.137 **	partial mediation	32.116%

Note: *p* < 0.05, **.

## Data Availability

The raw data supporting the conclusions of this article will be made available by the authors, without undue reservation.

## References

[1] Evans T.M., Bira L., Gastelum J.B., Weiss L.T., Vanderford N.L. (2018). Evidence for a mental health crisis in graduate education. Nat. Biotechnol..

[2] Levecque K., Anseel F., De Beuckelaer A., Van der Heyden J., Gisle L. (2017). Work organization and mental health problems in PhD students. Res. Policy.

[3] Rosseel Y. (2017). How are academic researchers doing? An international comparison based on a large-scale survey among academic researchers. Res. Eval..

[4] Woolston C. (2019). Graduate survey: A love–hurt relationship. Nature.

[5] Liu X., Liu L., Yang J. (2019). Mental health status and influencing factors among Chinese doctoral students. J. Affect. Disord..

[6] Zhang W., Liu Y. (2019). Mental health and its influencing factors among Chinese doctoral students: A cross-sectional study. PLoS ONE.

[7] Levecque K., Anseel F., Beuckelaer A.D. (2018). The role of workload and work–family conflict in job satisfaction and turnover intentions among Belgian academic researchers. J. Technol. Transf..

[8] LePine J.A., LePine M.A., Jackson C.L. (2004). Challenge and hindrance stress: Relationships with exhaustion, motivation to learn, and learning performance. J. Appl. Psychol..

[9] Mishra S., Srivastava A. (2021). Role of family support in the mental health of Indian research scholars. Curr. Psychol..

[10] Park H.I., Jacob A.C., Wagner S.H., Baiden M. (2014). Job control and burnout: A meta-analytic test of the Conservation of Resources model. Appl. Psychol..

[11] Jensen L.D., Jetten J. (2015). How social identity shapes the impact of stress on mental health: The good, the bad, and the ugly. Psychol. Inq..

[12] Sverdlik A., Hall N.C., McAlpine L., Hubbard K. (2018). The PhD experience: A review of the factors influencing doctoral students’ completion, achievement, and well-being. Int. J. Dr. Stud..

[13] Richardson T., Elliott P., Roberts R. (2017). The impact of tuition fees amount on mental health over time in British students. J. Public Health.

[14] Juniper B., Walsh E., Richardson A., Morley B. (2012). A new approach to evaluating the well-being of PhD research students. Assess. Eval. High. Educ..

[15] Tansley A.G. (1935). The use and abuse of vegetational concepts and terms. Ecology.

[16] Ulanowicz R.E., Goerner S.J., Lietaer B., Gomez R. (2009). Quantifying sustainability: Resilience, efficiency and the return of information theory. Ecol. Complex..

[17] Bronfenbrenner U. (1986). Ecology of the family as a context for human development: Research perspectives. Dev. Psychol..

[18] Bronfenbrenner U. (1979). The Ecology of Human Development: Experiments by Nature and Design.

[19] Evans G.W., Kim P. (2013). Childhood poverty, chronic stress, self-regulation, and coping. Child Dev. Perspect..

[20] Tudge J.R., Mokrova I., Hatfield B.E., Karnik R.B. (2009). Uses and misuses of Bronfenbrenner’s bioecological theory of human development. J. Fam. Theory Rev..

[21] Lazarus R.S., Folkman S. (1984). Stress, Appraisal, and Coping.

[22] Folkman S., Lazarus R.S. (1988). The relationship between coping and emotion: Implications for theory and research. Soc. Sci. Med..

[23] Hunter K.H., Devine K. (2016). Doctoral students’emotional exhaustion and intentions to leave academia. Int. J. Dr. Stud..

[24] Smith J.E., Wightman L.F. (2011). The impact of workload and research activity on the mental health of doctoral students in the sciences. High. Educ. Res. Dev..

[25] Hobfoll S.E. (1989). Conservation of resources: A new attempt at conceptualizing stress. Am. Psychol..

[26] Maslach C., Jackson S.E. (1981). The measurement of experienced burnout. J. Organ. Behav..

[27] Bandura A. (1977). Self-efficacy: Toward a unifying theory of behavioral change. Psychol. Rev..

[28] Cohen S., Wills T.A. (1985). Stress, social support, and the buffering hypothesis. Psychol. Bull..

[29] Rospenda K.M., Richman J.A., Nawyn S.J. (2017). Graduate student mental health: Needs assessment and utilization of counseling services. J. Coll. Stud. Dev..

[30] Eisenberg D., Golberstein E., Hunt J.B. (2009). Mental health and academic success in college. J. Econ. Perspect..

[31] Becker S.P., Cuccaro P., Drabick D.A. (2011). Parental support and adolescent depression: An examination of the mediating role of self-esteem and social competence. J. Abnorm. Child Psychol..

[32] Ream R.K., Gottfried M.A. (2019). Household wealth and adolescents’ social-emotional functioning in schools. Soc. Sci. Res..

[33] Cinamon R.G., Dan O. (2010). Parental attitudes toward preschoolers’ career education: A mixed-method study. J. Career Dev..

[34] Portes A. (1998). Social capital: Its origins and applications in modern sociology. Annu. Rev. Sociol..

[35] Cavanaugh M.A., Boswell W.R., Roehling M.V., Boudreau J.W. (2000). An empirical examination of self-reported work stress among US managers. J. Appl. Psychol..

[36] Haller A.O., Portes A. (1973). Status attainment process. Sociol. Educ..

[37] Zung W.W. (1965). A Self-Rating Depression Scale. Arch. Gen. Psychiatry.

[38] Xiao B., Song G.D. (2022). Association between Self-Efficacy and Learning Conformity among Chinese University Students: Differences by Gender. Sustainability.

[39] Xiao B., Song G.D. (2022). The Impact of Family Socioeconomic Status on Learning Conformity among Chinese University Students: Self-Efficacy as Mediating Factor. Sustainability.

[40] Smith J., Johnson M. (2018). The Psychological Loads of PhD Students in High-Intensity Academic Research Environments. High. Educ. Res. Dev..

[41] Wang L., Chen H. (2020). Research Pressure and Mental Health Among PhD Students: A Quantitative Analysis. J. Affect. Disord..

[42] Taylor S., Brown J. (2019). Stress Coping Theory and the Mental Health of PhD Students: A Qualitative Study. Stress Health.

[43] Johnson R.J., Wigley S. (2020). The Impact of Social Support on Mental Health Among Graduate Students: A Quantitative Analysis. J. Ment. Health Couns..

